# Guidelines for systematic reporting of sequence alignments

**DOI:** 10.1093/biomethods/bpaa001

**Published:** 2020-01-09

**Authors:** Mauno Vihinen

**Affiliations:** Department of Experimental Medical Science, Lund University, Lund, Sweden

**Keywords:** guidelines, systematic reporting of results, sequence alignment, reproducibility

## Abstract

Bioinformatics methods are increasingly needed and used to analyze and interpret extensive datasets many of which are produced by diverse high-throughput technologies. Unfortunately, it is quite common that published articles do not contain sufficient information to allow the reader to fully comprehend and repeat computational and other studies. Guidelines were developed for reporting studies and results from sequence alignment. Brief and concise checklist of required data items was compiled making it easy to provide necessary details. Implementation of the guidelines requires similar meticulous attitude toward details as other parts of publications. If the journal does not allow reporting full details in the main article, it can be provided in supplementary material. It is important to make the alignments available. Systematic and detailed description of bioinformatics analyses adds to the value of papers and makes it easier for the scientific community to evaluate, understand, verify, and extend the published articles and their results.

## Introduction

In many fields, experimental methods are used to produce huge amounts of data. Nucleic acid sequencing is a prime example—it is widely used throughout life science disciplines. To handle, store, share, and analyze this deluge of data numerous bioinformatics tools are required. The large body of recent medical and biological literature relies at least partly on computational analyses. It is apparent that all authors of these articles are not fully aware how to describe analyses and experiments performed on computers. This seems to apply to articles written both by bioinformaticians and others. The common rules for scientific literature apply also to these methods. However, it is quite common that the computational studies and experiments are not described in sufficient detail. There are several reasons for this, including space limitations in many journals, practices of replacing details by statements like “as previously described,” ignorance of the best practices and most importantly, lack of awareness of what is relevant.

Reporting of methods and results and their discussion has a number of functions in scientific literature. First, novel observations and interpretations are presented. Second, studies should contain detailed enough descriptions so that others could repeat them. Although this is the basic requirement for all kinds of reports, it is quite often neglected, at least partly. Problems with reproducibility of science have been raised in several fields. Results even in many prominent journals been irreproducible [1, 2]. Third, for the science to be reproducible, and self-correcting when necessary, it is important that the produced data are made available. Fourth, according to FAIR principles [3], research data should be made findable, accessible, interoperable, and reusable (https://www.force11.org/group/fairgroup/fairprinciples) and further global and open (GO FAIR, https://www.dtls.nl/fair-data/go-fair/). As scientists and clinicians, we should not “buy a pig in a poke,” i.e. demand all necessary details also when analyses are performed at commercial laboratories [4].

Problems with reproducibility in computational studies have been discussed [5, 6] along with recommendations e.g. for sharing data, software, workflows, citations, and others. One suggestion is a two-part solution to describe process and analysis metadata [7]. Big data poses special requirements, to handle such cases, methods like Nextflow [8] can be used.

To facilitate reproducibility, minimum information standards and criteria have been developed for reporting data from several types of experiments and distributed mainly in Fairsharing [9] (see https://fairsharing.org/). Some of them are widely used, especially when demanded by journals, as in the case of Minimum Information About a Microarray Experiment (https://fairsharing.org/FAIRsharing.32b10v) [10]. Minimum Information About Bioinformatics Investigation (https://fairsharing.org/FAIRsharing.28yec8) [11] provides basic reporting guidelines including the used algorithm, analysis protocol, used databases, resources, software, and (web) services.

Methods and results of bioinformatics studies are often inadequately described and documented in publications leaving readers wondering about important details and not being able to capture the message and reasoning of the authors. Since systematic guidelines have been missing for reporting sequence alignment studies, brief and concise checklist of required data items was produced. These rules are simple to follow and apply. It just requires the same attention to details as any other method used in research [12]. If the journal does not allow reporting full details in the main article, it can be provided in the supplementary material. Many details can also be included to tables, figures, and figure captions when they are needed to comprehend the results.

**Figure 1:**
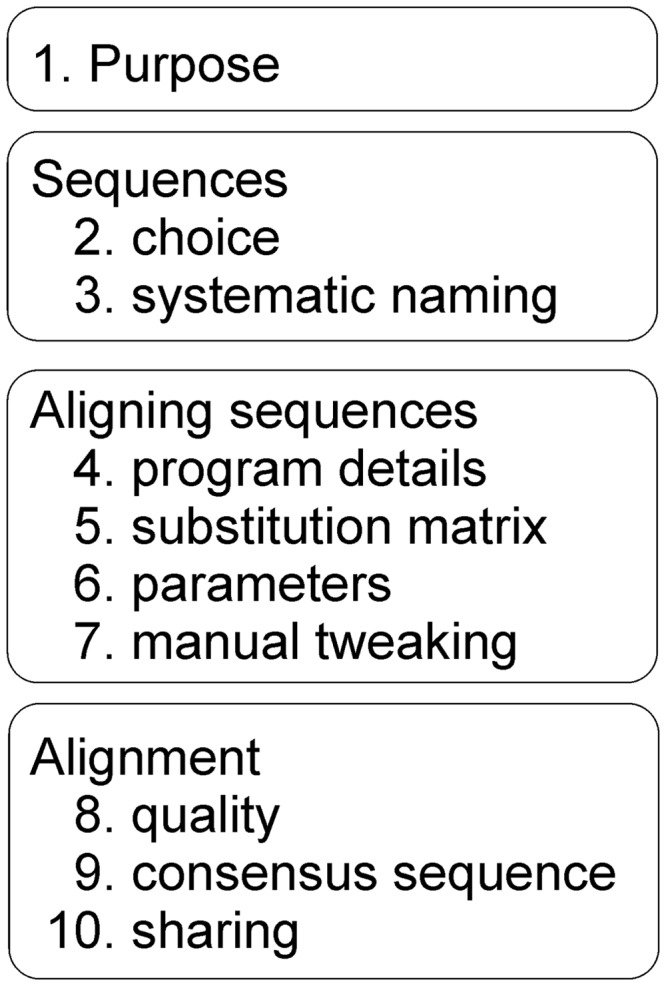
Items to be included in comprehensive description of sequence alignment.

The guidelines and the relations of the items are depicted in Fig. 1 and an example of their application in Supplementary File S1. There are four major categories: purpose of the study, analyzed sequences, alignment procedure, and reporting of the obtained alignment. It is necessary to follow all the items to provide a systematic and comprehensive description of alignments. These guidelines are applicable to all types of alignments.

### Describe the purpose of the alignment

Sequence alignment is a very widely used research method, since many different types of information can be obtained e.g. for interpretation of experimental results, about conserved and co-evolutionary sites, regions, motifs and domains and to help in diverse prediction tasks such as detection of homologs, protein secondary structural elements, functional characterization, variant interpretation including pathogenicity, etc. Alignment methods are instrumental also for evolutionary studies and protein homology modeling. The purpose of the alignment has to be mentioned as the analysis protocol may differ for different applications, e.g. whether one compares deoxyribonucleic acid (DNA) sequences to DNA sequences or against protein sequences (or vice versa*)*, or protein sequences to other protein sequences. Additional combinations include ribonucleic acid and DNA sequences.

Since in the case of proteins, alignments can be made either on the nucleotide or amino acid level, details for the analyzed level(s) have to be provided. Further necessary details contain whether the alignment is for two or multiple sequences.

### Justify the choice of included and excluded sequences

The choice of the sequences has to be carefully justified. Why were the included sequences chosen and why others were left out? These choices may have a significant impact on the obtained results. The data are important also if someone wants to repeat the analysis when new sequences become available to be able to apply the same criteria.

### List of sequences and entries, including database versions

The used sequences have to be properly documented, and unless Locus Reference Genomic (LRG) reference sequences [13] are used, there have to be version details for the files. LRG sequences are permanent entries and currently available for some human sequences. New ones can be obtained by making a request either to the European Bioinformatics Institute or the National Center for Biotechnology Information. Entries should be named in the same way and systematically throughout the article. Entries in some databases, such as UniProt, may contain several sequences. In such cases, the isoform identifier has to be provided.

### Reveal alignment program and its version

The program used for the alignment and its version has to be provided. Published methods have to be cited with proper reference and unpublished ones shared. In addition, mention the source (e.g. Uniform Resource Locator) of the software. If any changes are made to algorithms, scripts have to be published and shared, preferably in public repositories like GitHub.

### Define used substitution matrix

A substitution matrix is the central component for all sequence alignment programs and can affect the obtained results, thus details are needed. If any modifications have been made to published matrix, those have to be described. Provide a reference to the used matrix.

### Describe program parameters

It is mandatory to list all parameters and values used. Otherwise, the study is not reproducible. Justify the choice of the parameter values. Mentioning the default parameters is not sufficient, since they may vary between program versions and implementations.

### Describe manual modifications, if any

Sometimes it is necessary to make deviations and manual changes from standard procedures. When utilized, the changes have to be described in detail. Furthermore, these modifications have to be justified.

In general, manual interventions should be avoided since they are error-prone and difficult or impossible to reproduce e.g. with new data.

### Report quality of the alignment, with relevant measures

Quality indicators of the alignment have to be provided with adequate measures based on statistical analysis. Details depend on the used algorithm and on what it provides. In addition to similarity and/or identity score(s), an important aspect of the quality is the length of the alignment.

The quality measures will allow readers to evaluate the reliability of conclusions and claims made in the paper along with the actual alignment.

### Provide consensus sequence, if required for the application

A consensus sequence is an essential information for some alignment applications. It can be included to the alignment figure, usually on top or bottom of the aligned entries. Notation of the used symbols and e.g. the threshold for similarity has to be provided.

### Share the alignment

The alignment should be made available either in the article or in its supplementary materials, since otherwise it will be impossible e.g. to compare results from other studies or to judge the quality of the results. The details should preferentially be in computer-readable format to facilitate easy reuse.

## Discussion

Bioinformatics methods are increasingly needed and used to analyze and interpret extensive datasets, many of which are produced by diverse high-throughput technologies. Sequence alignments are popular and can reveal many details. Unfortunately, it is quite common that publications do not contain sufficient information. Guidelines were developed for reporting studies and results from sequence alignments. Supplementary File S1 shows how the guidelines can be applied in practice. Systematic and detailed descriptions add to the value of papers and make it easier for the scientific community to evaluate, understand, verify, and extend the publications and their results.

When reporting and interpreting alignments, authors have to consider the limitations also of their tools. Thus, authors have to know the quality of the data as computational predictions will be useless if the starting point is wrong or severely biased. Therefore, the choice of aligned sequences is important. They should be of high quality, preferably established reference sequences. Misinterpretations of bioinformatics results are common, the most common problem being too far-reaching conclusions based on data with weak support. Proper reporting of the studies will allow readers to pick such cases if peer review has failed in detecting the deficiencies.

Another frequent problem is that alignment results are often reported with the wrong terminology. For example, sequences are often erroneously claimed to be homologous. Sequence similarities mean homology only when the sequences have an evolutionary ancestor. For systematic homology-related terminology, there is ontology [14]. By using proper terminology and by following the guidelines articles readers will be able to understand performed studies and even cite them.

## Supplementary Material

bpaa001_Supplementary_Data
