# Supplementary material for: Guidelines for systematic reporting of sequence alignments
Source: Biol Methods Protoc. 2020 Jan 9;5(1):bpaa001. doi: 10.1093/biomethods/bpaa001 (PMC6994045; doi:10.1093/biomethods/bpaa001)
Supplement: bpaa001_Supplementary_Data [file bpaa001_supplementary_data.pdf]

Supplementary File 1

GUIDELINES FOR SYSTEMATIC REPORTING OF SEQUENCE ALIGNMENTS

Mauno Vihinen, Department of Experimental Medical Science, BMC B13, Lund University, SE-22 184 Lund, Sweden

Example of the application of guidelines to practice. These sentences could be part of the main text or be available in a supplement.

Purpose of the alignment

"The multiple sequence alignment of protein sequences of kinase domains of human TEC tyrosine kinase family members was made for homology modeling of family members without experimental structures."

Choice of sequences

"The longest isoforms of the human TEC family members were included to the analysis. These isoforms are by far the most prominent ones in immunological cell types."

Sequence entries

"The analysed sequence entries included P51813 for BMX, LRG_128 for BTK, Q08881-1 for ITK, P42680-1 for TEC, and P42681-1 for TXK. BTK sequence is from LRG the others from UniProtKB."

Alignment program details

"The alignment was performed on Clustal Omega program (Clustal O(1.2.4)) at https://www.ebi.ac.uk/Tools/msa/clustalo/.

Reference

Sievers, F., Wilm, A., Dineen, D., Gibson, T. J., Karplus, K., Li, W., Lopez, R., McWilliam, H., Remmert, M., Söding, J., Thompson, J. D., Higgins, D. G. Fast, scalable generation of

high-quality protein multiple sequence alignments using Clustal Omega. Mol Syst Biol. 2011 7:539."

Substitution matrix

"Substitution matrix was of Gonnet et al. (Gonnet, G. H., Cohen, M. A., Benner, S. A. Exhaustive matching of the entire protein sequence database. Science. 1992, **256,**1443-1445."

Program parameters

"The used parameters were: Output guide tree, false; Output distance matrix, false; Dealign input sequences, false; mBed-like clustering guide tree, true; mBed-like clustering iteration, true; Number of iterations, 0; Maximum guide tree iterations, -1; Maximum HMM iterations, -1; Output alignment format, clustal_num; Output order, aligned; Sequence type, protein. The sequences are highly similar to each other and thus changes of parameters do not change the alignment."

Information about manual modifications

"No manual modifications were made to the alignment."

Alignment quality measures

"The alignment covers 99.2-100% of the sequence lengths. The program provides also a percent identity matrix for pairwise comparisons:

1: BMX	100.00	65.50	58.53	59.85	61.48
2: BTK	65.50	100.00	57.36	62.40	60.31
3: ITK	58.53	57.36	100.00	65.50	64.98
4: TEC	59.85	62.40	65.50	100.00	68.48
5: TXK	61.48	60.31	64.98	68.48	100.00

Consensus sequence

"Consensus sequence indicating 70% sequence identity is shown under the alignment"

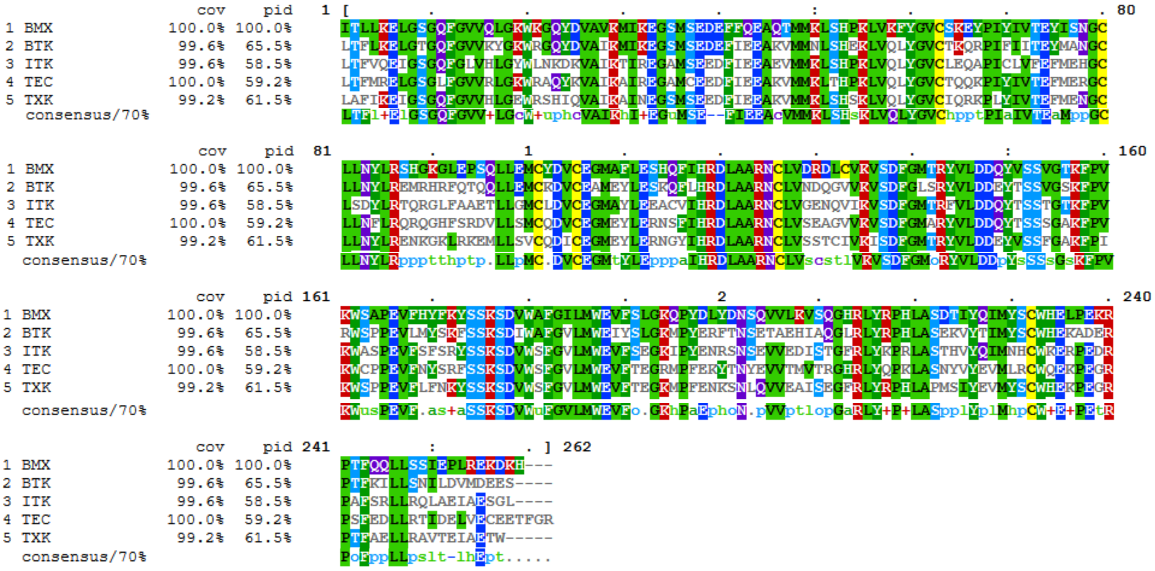

Sharing the alignment

“The alignment is available in Figure X”.

It is either part of the main paper or included as supplementary material.

```
BMX       ITLLKELGSGQFGVVQLGKWKGQYDVAVKMIKEGSMSEDEFFQEAQTMMKLSHPKLVKFY        60
BTK       LTFLKELGTGQFGVVKYGKWRGQYDVAIKMIKEGSMSEDEFIEEAKVMMNLSHEKLVQLY        60
ITK       LTFVQEIGSGQFGLVHLGYWLNKDKVAIKTIREGAMSEEDFIEEAEVMMKLSHPKLVQLY        60
TEC       LTFMRELGSGLFGVVRLGKWRAQYKVAIKAIREGAMCEEDFIEEAKVMMKLTHPKLVQLY        60
TXK       LAFIKEIGSGQFGVVHLGEWRSHIQVAIKAINEGSMSEEDFIEEAKVMMKLSHSKLVQLY        60
          :::::*:*:* **:*: * *  : .**:* *.**:*.*::*::**:.**:*:* ***::*

BMX       GVCSKEYPIYIVTEYISNGCLLNYLRSHGKGLEPSQLLEMCYDVCEGMAFLESHQFIHRD        120
BTK       GVCTKQRPIFIITEYMANGCLLNYLREMRHRFQTQQLLEMCKDVCEAMEYLESKQFLHRD        120
ITK       GVCLEQAPICLVFEFMEHGCLSDYLRTQRGLFAAETLLGMCLDVCEGMAYLEEACVIHRD        120
TEC       GVCTQQKPIYIVTEFMERGCLLNFLRQRQGHFSRDVLLSMCQDVCEGMEYLERNSFIHRD        120
TXK       GVCIQRKPLYIVTEFMENGCLLNYLRENKGKLRKEMLLSVCQDICEGMEYLERNGYIHRD        120
          *** :. *: :: *:: .*** ::**      :  . ** :* *:**.* :**    :***

BMX       LAARNCLVDRDLCVKVSDFGMTRYVLDDQYVSSVGTKFPVKWSAPEVFHYFKYSSKSDVW        180
BTK       LAARNCLVNDQGVVKVSDFGLSRYVLDDEYTSSVGSKFPVRWSPPEVLMYSKFSSKSDIW        180
ITK       LAARNCLVGENQVIKVSDFGMTRFVLDDQYTSSTGTKFPVKWASPEVFSFSRYSSKSDVW        180
TEC       LAARNCLVSEAGVVKVSDFGMARYVLDDQYTSSSGAKFPVKWCPPEVFNYSRFSSKSDVW        180
TXK       LAARNCLVSSTCIVKISDFGMTRYVLDDEYVSSFGAKFPIKWSPPEVFLFNKYSSKSDVW        180
          ********.    :*:****::*:****:*.** *:***::*. ***: : ::*****:*

BMX       AFGILMWEVFSLGKQPYDLYDNSQVVLKVSQGHRLYRPHLASDTIYQIMYSCWHELPEKR        240
BTK       AFGVLMWEIYSLGKMPYERFTNSETAEHIAQGLRLYRPHLASEKVYTIMYSCWHEKADER        240
ITK       SFGVLMWEVFSEGKIPYENRSNSEVVEDISTGFRLYKPRLASTHVYQIMNHCWKERPEDR        240
TEC       SFGVLMWEVFTEGRMPFEKYTNYEVVTMVTRGHRLYQPKLASNYVYEVMLRCWQEKPEGR        240
TXK       SFGVLMWEVFTEGKMPFENKSNLQVVEAISEGFRLYRPHLAPMSIYEVMYSCWHEKPEGR        240
          :**:****::: *: *::    * :..   :: * ***:*:**   :* :*  **:*   : *

BMX       PTFQQLLSSIEPLREKDKH---   259
BTK       PTFKILLSNILDVMDEES----   258
ITK       PAFSRLLRQLAEIAESGL----   258
TEC       PSFEDLLRTIDELVECEETFGR   262
TXK       PTFAELLRAVTEIAETW-----   257
          *:*  **  :  : :
```
